# Interstitial high-dose rate brachytherapy as boost for anal canal cancer

**DOI:** 10.1186/s13014-014-0240-4

**Published:** 2014-11-06

**Authors:** Alexander Tuan Falk, Audrey Claren, Karen Benezery, Eric François, Mathieu Gautier, Jean-Pierre Gerard, Jean-Michel Hannoun-Levi

**Affiliations:** Department of Radiation Therapy, Antoine Lacassagne Cancer Center, 33, Avenue de Valombrose, 06189 Nice, Cedex France; University of Nice Sophia-Antipolis, Nice, France; Departement of Medical Oncology, Antoine Lacassagne Cancer Center, Nice, France

**Keywords:** Brachytherapy, Anal canal cancer, High-dose rate, Boost, Radiotherapy

## Abstract

**Aim:**

To assess clinical outcomes of patients treated with a high-dose rate brachytherapy boost for anal canal cancer (ACC).

**Methods:**

From August 2005 to February 2013, 28 patients presenting an ACC treated by split-course external beam radiotherapy (EBRT) and HDR brachytherapy with or without chemotherapy in a French regional cancer center in Nice were retrospectively analyzed.

**Results:**

Median age was 60.6 years [34 – 83], 25 patients presented a squamous cell carcinoma and 3 an adenocarcinoma; 21 received chemotherapy. Median dose of EBRT was 45 Gy [43.2 – 52]. Median dose of HDR brachytherapy was 12 Gy [10 - 15] with a median duration of 2 days. Median overall treatment time was 63 days and median delay between EBRT and brachytherapy was 20 days. Two-year local relapse free, metastatic free, disease free and overall survivals were 83%, 81.9%, 71.8% and 87.7% respectively. Acute toxicities were frequent but not severe with mostly grade 1 toxicities: 37% of genito-urinary, 40.7% of gastro-intestinal and 3.7% of cutaneous toxicities. Late toxicities were mainly G1 (43.1%) and G2 (22%). Two-year colostomy-free survival was 75.1%, one patient had a definitive sphincter amputation.

**Conclusion:**

High-dose rate brachytherapy for anal canal carcinoma as boost represents a feasible technique compared to low or pulsed-dose rate brachytherapy. This technique remains an excellent approach to precisely boost the tumor in reducing the overall treatment time.

## Introduction

Anal canal carcinoma (ACC) is a rare disease with an incidence rate of less than 10 cases per 1 000 000 habitants in Europe [1], which has been considered as a life deteriorating even when cured because of sphincter amputation caused by historical surgical treatment. The impact on quality of life has pushed clinicians to consider new treatment ways. Conservative treatments for sphincter preservation in ACC have become a standard, even for large tumors. Gold standard treatment consists of concomitant radiotherapy and chemotherapy with Mitomycin and 5-Fluorouracil [2-4]. Clinical target volume consists of the gross tumor volume and the loco-regional lymph nodes with a dose of 45 to 50 Gy with a sequential boost delivered by either external beam radiation therapy (EBRT) or by interstitial brachytherapy (BCT) [5,6]. All attempts to find a better standard of chemotherapy have failed [7]. However, recent development of radiotherapy technique have permitted better care and new treatment strategies for ACC [8,7].

There is no agreement for the boost technique. Standard brachytherapy technique remains based on LDR or PDR [9,10]. However as proposed for numerous tumors (prostate [11] and cervical cancer [12]) HDR brachytherapy appears to be more and more used mainly because of radioprotection considerations, dose distribution optimization and shorter treatment time [13]. Currently, there is few available data focusing on high-dose rate brachytherapy for ACC. This study was aimed to assess clinical outcomes of patients treated with a HDR brachytherapy boost for anal canal cancer.

## Material and methods

### Patient features

This study retrospectively analyzed the clinical outcome of patients with histology-proven ACC treated by split-course radiotherapy and HDR brachytherapy with or without chemotherapy in the Centre Antoine Lacassagne, a regional cancer center in Nice, France. The study design and analysis was approved by the local institutional ethic comity of Antoine Lacassagne Cancer Center.

From August 2005 to February 2013, 28 patients were retrospectively analyzed. All the patients had a clinical exam and follow-up done by trained physicians of the center. According to local rules, patients underwent a digital rectal examination with a dated schema, anuscopy, computed-tomography scan, magnetic resonance imaging and endorectal ultrasonography. PET-scan was performed at the discretion of the physician. Tumors were staged using the UICC-cTNM classification (2002 – 6^th^ edition). All the patients presenting with a tumor involving no more than 2/3 of the anal canal circumference were eligible for brachytherapy boost. Due to a high risk of necrosis/stenosis, circumferential tumor was considered as a definitive exclusion criteria for brachytherapy.

### Treatment features

#### Concomitant radio-chemotherapy

The first part of the treatment consisted of EBRT with or without concurrent chemotherapy. Radiotherapy delivered a total dose of 45 to 46 Gy in 25 or 23 fractions based on a 3-dimensional conformational technique with or without intensity modulated. The dose was delivered to the ICRU point (International Commission on Radiation Units Measurements) using high-energy X-photons (>10 MV). The clinical target volume (CTV) was the whole pelvis. The planning target volume (PTV) was defined as a 1 cm margin around the CTV in all directions. Inclusion of the inguinal nodes was set at the discretion of the radiation oncologist.

Patients who received concomitant chemotherapy were administrated Mitomycin C - 5FU or Cisplatin - 5FU.

#### HDR brachytherapy boost

Brachytherapy boost was performed unless patients could not undergo general anesthesia or presented with a tumor invasion >2/3 of the circumference. HDR brachytherapy was performed after the patient recovered from perineal dermatitis. After a 2 day fiber free diet and enema the day before and one-hour before procedure. A digital exam and anuscopy were performed under general anesthesia in order to evaluate the tumor response after radio-chemotherapy. A Foley catheter was first introduced into the bladder, then, needles (Sharp Needles™; Nucletron, an Elekta company, Elekta AB, Stockholm, Sweden) were implanted under general anesthesia according to the pre-treatment target volume (based on the initial schema) respecting a minimal distance of 4 to 5 mm from the needles to the anal canal mucosa. We used a dedicated circular perineal template punched by a total of 10 holes (every 12 mm) allowing to keep the implanted needles equidistant and parallel. A plastic tube (20 mm in external diameter) was placed into the anal canal and fixed to the perineal template which was finally sutured to the skin. After recovery, post-implant planning CT-scan was performed in the radiation oncology department for treatment planning purposes. CTV was delineated (anal canal initial tumor) using a 10 mm in diameter pearl.

**Figure 1:**
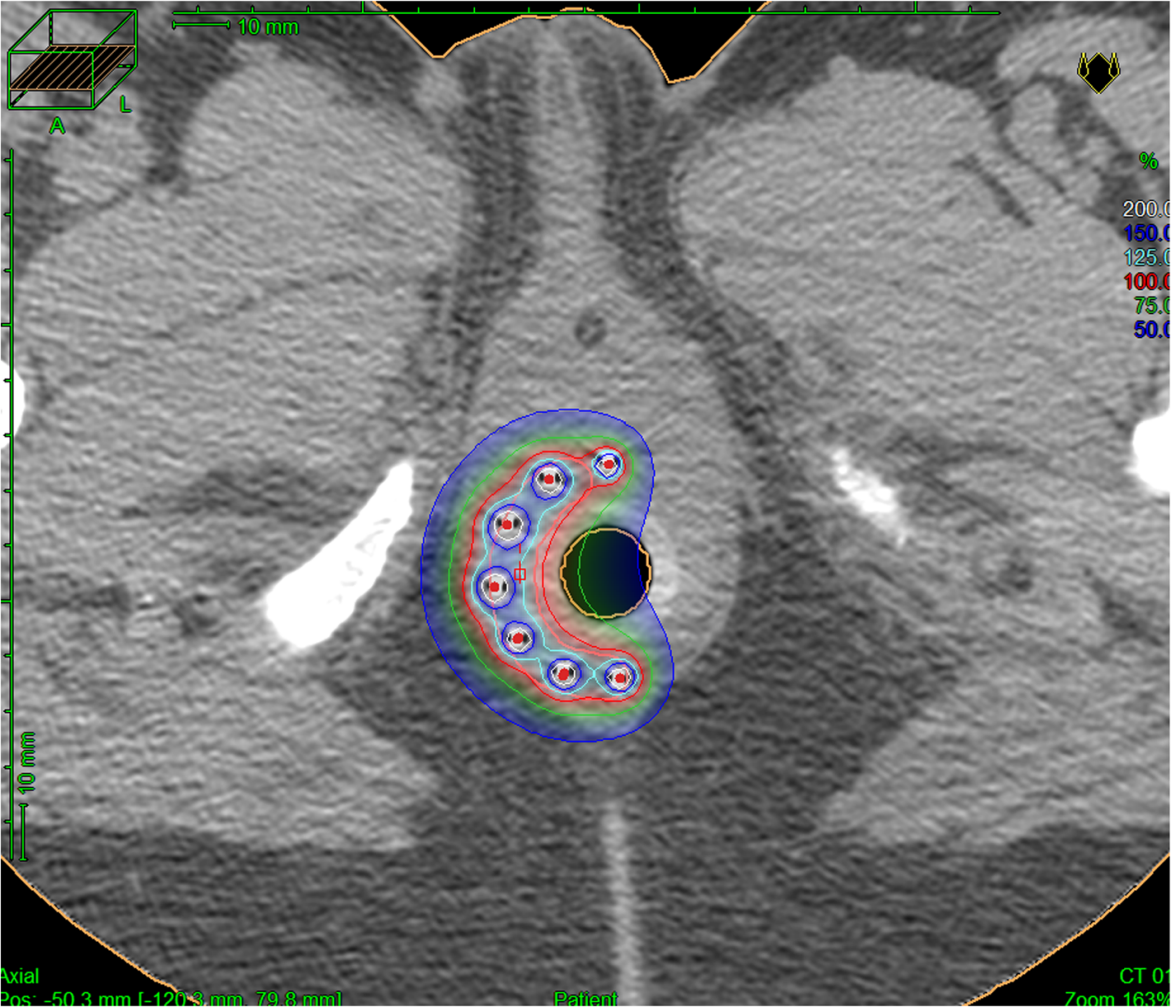
**Dose distribution analysis on the post-implant CT-scan.**

Regarding the prescribed dose, our standard protocol was based on the result of the digital rectal exam performed under general anesthesia before the implant. In case of complete clinical response, a total dose of 12 Gy in 3 fractions over 24 hours (EQD2_αβ3_ = 17 Gy/EQD2_αβ10_ = 15 Gy) was prescribed (1 fraction delivered the day of implant and 2 fractions at least 6 hours apart the day after). In case of partial clinical response, a total dose of 15 Gy in 3 fractions over 24 hours (EQD2_αβ3_ = 24 Gy/EQD2_αβ10_ = 19 Gy) was prescribed. Dose-volume adaptation was manually achieved using graphical optimization (OncentraBrachy, Nucletron, an Elekta company, Elekta AB, Stockholm, Sweden) by dwell location and time variation (Figure 1). During the treatment, the patient was treated in his bed and hospitalized in a non-shielded room. After the last irradiation session, the needles were removed and the patient left the hospital the day after.

Regarding dosimetric results, D90 (dose delivered to 90% of the CTV) and D100 were reported as well as V100 (volume receiving 100% of the prescribed dose), V150 and V200. DHI (Dose Homogeneity Index: [V100-V150]/V100) was also calculated.

### Follow-up and evaluation

Patients were followed-up one month after HDR brachytherapy then, every semester with radiation oncologist and gastro-enterologist alternatively by clinical examination, endorectal ultrasonography and MRI if necessary. Local (LRFS) and metastatic (MRFS) relapse-free survivals as well as disease free (DFS), overall (OS) and colostomy-free (CFS) survivals were analyzed. Early and late toxicities rates were graded using the NCI-Common criteria version 4.0 [14].

### Statistical analysis

All the data was analyzed using SPSS 20.0 (IBM Corporation). Overall survival was defined as the delay between date of diagnostic and the date of death. Relapse was defined as the delay between the date of diagnostic and the date of relapse. These statistics were estimated and represented graphically using Kaplan-Meier method. Patients were censored at the moment of their death or their last follow-up.

## Results

Twenty-eight patients (pts) were analyzed in this study; patient characteristics are reported in Table 1. Median follow-up was 27.5 [4–98 months] and median age was 60.6 years [34–83 years]. Median initial tumor size was 3 cm [1–6 cm] and, using cTNM classification, the large majority of the patients were classified as T1/2 (90%). Histologic subtypes were as follow: 25 pts presented a squamous cell carcinoma and 3 pts an adenocarcinoma. Two patients were HIV positive. Twenty-one patients (71.4%) received chemotherapy (17 pts: 5FU/Mitomycin-C and 4 pts received 5FU/Cisplatin). Patients received a median dose of EBRT of 45 Gy [43.2–52 Gy] over a median EBRT treatment time of 37 days [32–52 days]. Median dose for HDR brachytherapy was 12 Gy [10–15 Gy]. Median duration of brachytherapy was 2 days [2,3]. Median overall treatment time was 63 days [38–74 days] and median delay between EBRT and HDR brachytherapy was 20 days [4–63 days]. Dosimetric data are reported in Table 2. Briefly, median CTV was 22.3 cc [8.6 - 46.7 cc], V100 was 96% [58 – 100%] and median DHI was 0.55 [42 – 99].

**Table 1:** **Patient, tumor and treatment features**

**Items**	**N/Median**	**Interval/%**
Age (years)	60	[34 – 83]
Sex ratio (F/M)	3	
VIH Positive	2	7.1
Tumor stage		
T1	5	17.9
T2	20	71.4
T3	2	7.1
T4	1	3.6
Lymph node status		
N0	25	89.3
N1	2	7.1
N2	1	3.6
Histologic type		
SCC	25	89.3
ADC	3	10.7
EBRT		
Chemotherapy	20	71.4
Inguinal irradiation	20	71.4
Total dose (Gy)	45	[43.2 – 52]
HDR BT		
d/f (Gy)	4	[3 – 5]
# of fractions	3	[2 – 6]
Total dose (Gy)	12	[10 – 15]
# of needles	4	[4 – 7]
TI EBRT/BT (days)	20	[4 – 63]
OTT (days)	63	[38 – 74]

SCC: squamous cell carcinoma; ADC: adenocarcinoma; EBRT: external beam radiation therapy; HDR BT: high-dose rate brachytherapy; d/f: dose per fraction; TI EBRT/BT: time interval between external beam radiation therapy and brachytherapy; OTT: overall treatment time.

**Table 2:** **Dosimetric data**

**Data**	**Median**	**Interval**
CTV (cc)	22.3	[8.6 - 46.7]
D90%	108	[32 – 117]
EQD2 αβ10 (Gy)	15.1	[5.5 – 20.6]
EQD2 αβ3 (Gy)	18.1	[6.9 – 26.4]
D100%	77	[20 – 95]
EQD2 αβ10 (Gy)	10.9	[3.4 – 14.4]
EQD2 αβ3 (Gy)	13.3	[4.3 – 18.5]
V100		
%	96	[58 – 100]
cc	18	[8.1 – 45.8]
V150		
%	37	[26 – 57]
cc	7.2	[2.5 – 26.6]
V200		
%	19	[10 – 26]
cc	3.4	[1.2 – 11.7]
DHI	0.58	[0.42 – 0.99]

CTV: Clinical target volume; D90: dose delivered to 90% of the CTV; EQD2 αβ10: equivalent dose at 2 Gy per fraction for αβ10 (tumor); EQD2 αβ3: equivalent dose at 2 Gy per fraction for αβ3 (normal tissues); V100: volume which received 100% of the prescribed dose; V150: volume which received 150% of the prescribed dose; V200: volume which received 200% of the prescribed dose; DHI: dose homogeneity index.

**Figures 2:**
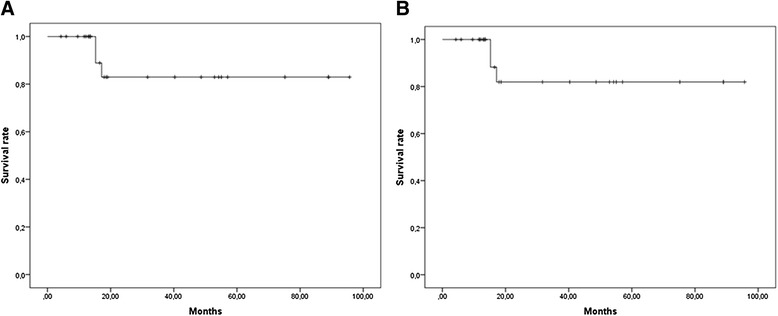
**Kaplan-Meier curves for local disease free survival (A) and metastatic free survival (B).**

**Figures 3:**
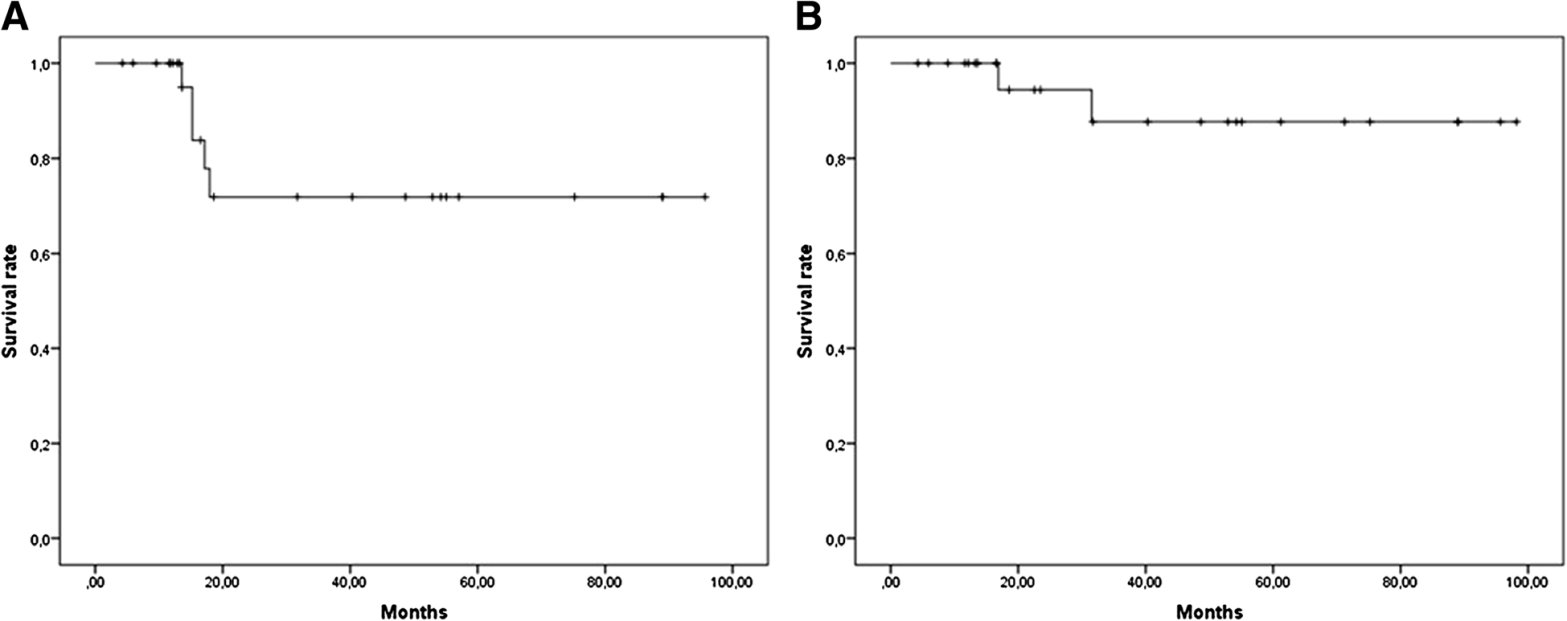
**Kaplan-Meier curves for disease free survival (A) and overall survival (B).**

In terms of clinical outcomes, 2-years LRFS and MRFS rates were 83% [Standard Error (SE) 7.8%] and 81.9% [SE 9.5%] respectively (Figure 2A and 2B).Two-year DFS and OS rates were 71.8% [SE 10.7%] and 87.7% [SE 8.2%] (Figure 3A and 3B) respectively.

**Figure 4:**
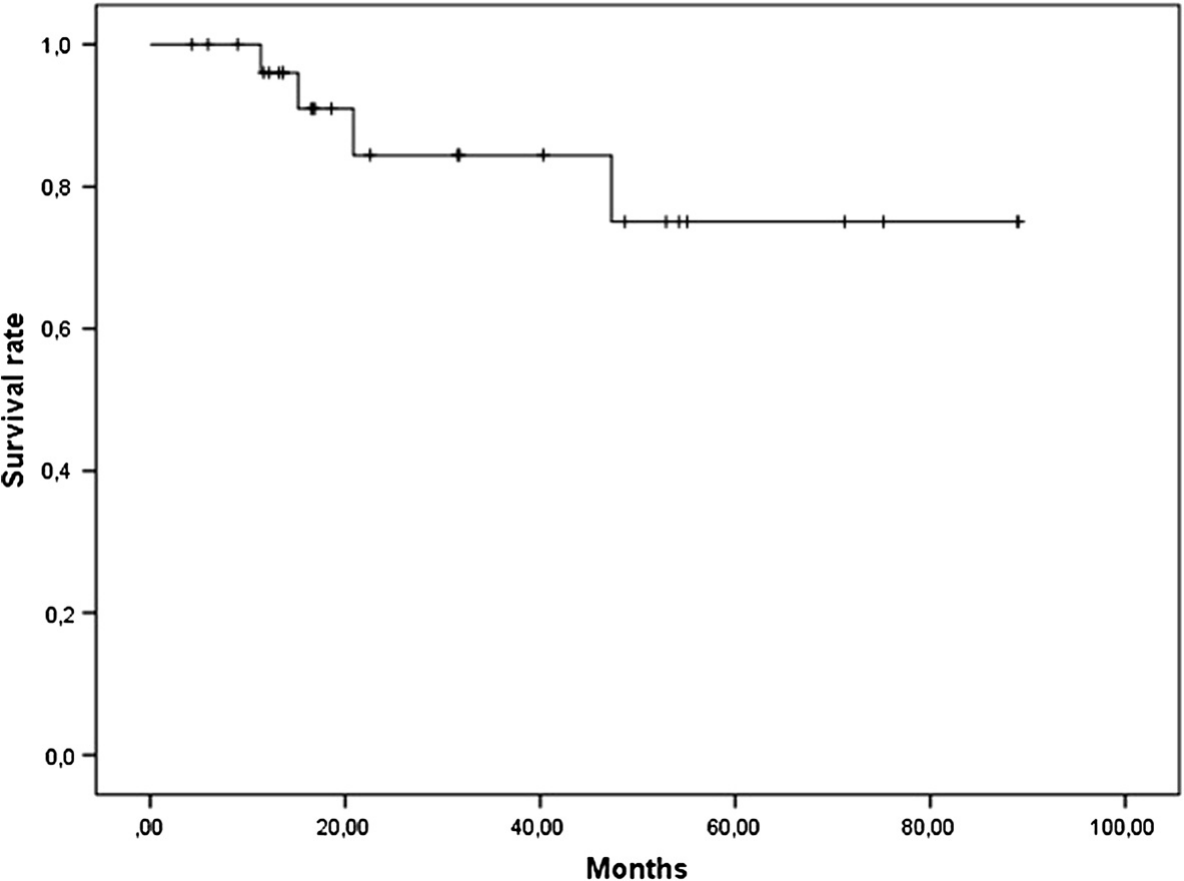
**Colostomy-free survival.**

Acute toxicities following HDB (<2 months) were frequent but not severe. Indeed, genito-urinary (GU), gastro-intestinal (GI) and cutaneous toxicities were always grade 1 (G1) and occurred in 37%, 40.7% and 3.7% respectively. Late toxicities were mainly G1 (43.1%) and G2 (22%). GI complications were: rectal bleeding (16.5%), perineal pain (13.2%), telangiectasia (13.2%), diarrhea (9.9%), rectal mucus (9.9%), constipation (3.3%), abdominal pain (3.3%) and rectal pain (3.3%). GU complications were: frequent urination (13.2%), incontinence (6.6%), hematuria (3.3%), urgency (3.3%). Two women (7.1%) presented G3 late complications: the first (HIV-positive) with grade 2 rectal bleeding and grade 3 anal ulceration, with complete recovery after 5 years; the second with a grade 3 anal necrosis managed with colostomy. Two-year CFS was 75.1% [SE 11.6%] (Figure 4). Among the four patients (14.3%) who underwent a colostomy, one was associated with a definitive sphincter amputation.

## Discussion

Brachytherapy plays a key role in the management of ACC as boost after a first course of EBRT with or without concomitant chemotherapy [5]. From now, brachytherapy for ACC used mainly LDR or PDR. However, due to the possibilities of dose distribution optimization, but also radioprotection and cost-effective considerations, HDR brachytherapy gains, step by step, in respectability. While this technique represents a standard treatment for cervical [12] and prostate cancers [11], there is very few available data concerning ACC.

Optimization of dose distribution used with PDR/HDR brachytherapy represents in general a real advantage compare to LDR brachytherapy. However, specifically for ACC brachytherapy, because of the very proximal situation of the CTV in regards to the perineal template used (optimal needle guidance), the impact of dose optimization is maybe less important although adequate coverage of the anal mucosa (avoiding overdose area) appears more feasible. Classical dosimetric parameters used for HDR brachytherapy (CTV, D90, D10, V100, V150 and V200) can be used for ACC. Kapoor et al. [15] recently reported the results of 16 patients treated by radio-chemotherapy and HDR brachytherapy and presented dosimetric data with a mean DHI of 0.83 [0.55–0.98]. In our study, we reported a median DHI of 0.58.

In terms of clinical outcomes, specifically regarding OS, CFS and toxicity ≥ G3, it seems that our results are comparable to previous ACC brachytherapy series using LDR, PDR or HDR (Table 3). For LCR, our series reported a rate of 83% with a 27.5 median FU. This result is quite comparable to those presented by the other HDR studies [16-19,15]. We reported a 2-year OS rate of 87.7%. A historical clinical study published by Papillon et al. [9] focusing on LDR brachytherapy showed a 69.2% OS rate at 3 years. More recent PDR studies from Gerard et al. [20] and Bruna et al. [21] found respectively an OS rate of 100% at 1 year and 90% at 2 years. Lòpez Guerrera et al. [22] presented a mixed study of LDR and PDR brachytherapy with a 2-year OS rate of 87%. These results are similar to those observed with external radiotherapy (Table 4).

**Table 3:** **Comparative overview of the literature focusing on brachytherapy boost for anal canal carcinoma according to the brachytherapy dose-rate**

**Authors (Year)**	# pts*	**Median FU (months)**	**Dose Rate**	**Median BT dose (Gy)**	**LR (%)**		**OS (%)**	**Colostomy**		**Late toxicity ≥ G3 [%]**
					**LRR (%)**	**LCR (%)**		**CR %**	**CFS %**	
Peiffert et al. (1997) [16]	101/118	72	LDR	21.5	N/A		60 (5 y)	9	-	13.5
Papillon et al. (1989) [17]	221	> 36	LDR	20 to 30	N/A		69.2 (3 y)	2.7	-	N/A
Wagner et al. (1994) [18]	96	35.5 to 51.7**	LDR	20.2**	16.6	-	64 (5 y)	N/A		9.25
Gerard et al. (1999) [19]	19	N/A	PDR	10 to 25	5,2	-	100 (1 y)	0	-	0
Bruna et al. (2006) [20]	71	28.5	PDR	17.8**	N/A	90 (2y)	-	89 (2 y)	N/A	
Roed et al. (1996) [10]	17	11.3	PDR	28.85	23.5	-	N/A	47	-	N/A
Kapp et al. (2001) [21]	39	31	HDR	6 to 12***		81 (3 y)	80 (3 y DSS)	-	78 (3 y)	7.6
						76 (5 y)	76 (5 y DSS)		73 (5 y)	
Vordermark et al. (2001) [5]	20	52.8**	HDR	5 to 12	5	-	84 (5 y)	-	68.9 (5 y)	N/A
Doniec et al. (2006) [23]	50	34	HDR	8 to 12	N/A		74 (5 y)	4	-	N/A
Oehler-Jänne et al. (2007) [24]	34	60**	HDR	14	10.3 (5y)	-	66 (10 y)	15	-	N/A
Saarilahti et al. (2008) [25]	29/59	51	HDR	12	N/A		N/A	N/A		N/A
Kapoor et al. (2013) [15]	16	41	HDR	18 (6f)	-	87.5 (2 y)	N/A	N/A		0
				21 (7f)				N/A		
Falk et al. (2014)	28	27.5	HDR	12 (3f)	-	83 (2 y)	87.7	-	75.1 (2 y)	7.1

*If ratio, number of patients treated by brachytherapy boost/total number of patients treated.

**Mean.

***6 Gy during the EBT period and 6 Gy after EBT in case of partial response.

# pts: number of patients; FU: follow-up; BT: brachytherapy; LR: local recurrence; LRR: local recurrence rate; LCR: local control rate; OS: overall survival; CR: colostomy rate; CFS : colostomy free survival; LDR: low-dose rate brachytherapy; PDR: pulsed dose rate brachytherapy; HDR: High-dose rate brachytherapy; NA: non-applicable; DSS: disease specific survival.

**Table 4:** **Overview of the literature focusing on external radiotherapy for anal canal carcinoma**

**Authors (Year)**	**EBT type**	**# pts**	**Median FU (months)**	**Median EBRT dose (Gy)**		**LR**		**OS (%)**	**Colostomy**		**Late toxicity ≥ G3 [%]**
				**Initial**	**Boost**	**LRR (%)**	**LCR (%)**		**CR %**	**CFS %**	
Northover et al. (2010) [5]	2DRT	290	157	45	N/A	57.1 (5 y)	33.7 (5 y)	53 (5 y)		36.8 (5 y)	N/A
Northover et al. (2010) [5]	2DRT + CT	295	157	45	N/A	32.3 (5 y)	46.6 (5 y)	58.1 (5 y)		46.9 (5 y)	N/A
Ajani et al. (2008) [23]	2DRT MMC-arm	324	30	45	10-14	25 (5 y)		84% (5 y)	10% (5y)		11%
Kachnic et al. (2013) [24]	IMRT	43	24	42*	50.4*		95% (2 y)	94% (2 y)		90% (2 y)	7%
				50.4**	54**						

*For T1-T2 : 42 Gy with 50.4 Gy integrated boost on the tumor volume.

**For T3-T4 : 50.4 Gy with 54 Gy integrated boost on the tumor volume.

EBT: external beam radiation therapy; # pts: number of patients; FU: follow-up; LR: local recurrence; LRR: local recurrence rate; LCR: local control rate; OS: overall survival; CR: colostomy rate; CFS : colostomy free survival; NA: non-applicable; DSS: disease specific survival; 2DRT: 2 dimension radiation therapy; CT: chemotherapy; MMC: Mitomycin-C; IMRT: intensity modulated radiation therapy; N/A: not applicable.

Our patients presented frequent grade 1–2 toxicities but grade 3 toxicities only occurred for 7.1% of patients. Severe toxicities (≥ G3) were reported in 0 to 13.5% of patients in LDR/PDR studies [9,25,26,10,20,27] and in 0 to 7.6% in HDR studies [28,29]. Only 2 patients presented severe grade 3 toxicities, one of them with an HIV-positive status which could account for higher toxicities [30]. Compared to our results, Kapoor *et al*. [15] reported no grade 3 toxicities with a similar LCR. This observation could be explained by the difference in terms of DHI with a less homogeneous implant in our cohort. On another hands, the total number of HDR brachytherapy fractions ranged between 6 and 7 in the Kapoor *et al*. study while in our cohort, the patients were treated with only 3 fractions (potential reduction of the hospitalization time). Nevertheless, our CFS rate (75.1%) remains comparable to other published data on LDR, PDR and HDR brachytherapy [16,28].

Due to its design, we weren’t able to access quality of life. To our knowledge, there is no comparative assessing quality of life for brachytherapy boost. External radio chemotherapy seems to be associated with good quality of life with 77% patients being satisfied of their body [31]. Recent international guidelines have encouraged the study and evaluation of sexual, urinary and sphincter dysfunction as the data are scarce [32].

This study presented limitations. Data collection was retrospective and monocentric. Median follow-up was short (27.5 months). We didn’t directly compare our data to low or pulsed-dose rate brachytherapy or EBRT boost.

HDR brachytherapy has advantages compared to low or PDR brachytherapy. It permits less radiation exposure for the medical staff and could provide better dose-distribution compared to LDR brachytherapy. Patients can be hospitalized in a non-shielded room allowing visits of the family (forbidden in case of LDR or PDR brachytherapy) with a potential improvement of psychological experience. Furthermore, with only one after-loader HDR machine, is it possible to treat many patients per day while for LDR or PDR, the number of treated patients per day will be strongly correlated to the number of shielded-rooms (potential cost-effective impact). To our knowledge, there are no prospective studies evaluating HDR brachytherapy for anal canal cancer boost after sequential EBRT. Further data needs to be collected and maybe even compared to LDR/PDR brachytherapy.

## Conclusion

High-dose rate for anal canal carcinoma as boost represents a feasible technique compared to low or pulsed-dose rate brachytherapy. This technique remains an excellent approach to precisely boost the tumor in reducing the overall treatment time while high-dose rate is more and more used in the frame of brachytherapy. Currently simultaneous-integrated boost (SIB) in case of intensity-modulated EBRT represents a challenging therapeutic option which could be compared to HDR brachytherapy for ACC boost.

### Consent

Written informed consent was obtained from the patient for the publication of this report and any accompanying images.
